# Impact of placental malaria on maternal, placental and fetal cord responses and its role in pregnancy outcomes in women from Blue Nile State, Sudan

**DOI:** 10.1186/s12936-021-03580-x

**Published:** 2021-01-09

**Authors:** Samia Omer, Clara Franco-Jarava, Ali Noureldien, Mona Omer, Mutasim Abdelrahim, Israel Molina, Ishag Adam

**Affiliations:** 1 Department of Immunology and Biotechnology, Tropical Medicine Research Institute, Khartoum, Sudan; 2 Immunology Department, Vall d Hebron University Hospital, Barcelona, Spain; 3 Department of Microbiology and Parasitology, Faculty of Medicine, University of Khartoum, Khartoum, Sudan; 4 Bioscience Research Institute, Ibn Sina University, Khartoum, Sudan; 5 Ed-Damazin Hospital, Blue Nile State Ministry of Health, Ed-Damazin, Sudan; 6 Infectious Diseases Department, Vall d Hebron University Hospital, Barcelona, Spain; 7 Department of Obstetrics and Gynecology, Unaizah College of Medicine and Medical Sciences, Qassim University, Unaizah, Kingdom of Saudi Arabia

**Keywords:** Placental malaria, Cytokines, Birth weight, Blue Nile State Sudan

## Abstract

**Background:**

The sequestration of *Plasmodium falciparum* infected cells in the placenta results in placental malaria (PM). It activates the mother's immune cells and induces secretion of inflammatory cytokines, which might influence pregnancy outcomes. This study aims to investigate the cytokines (levels IL-4, IL-6, IL-10, IL-17A, and INF γ) in maternal peripheral, placental, and umbilical cord blood in response to PM and the extent to which this may influence maternal haemoglobin levels and birth weight.

**Methods:**

A total of 185 consenting Sudanese women from Blue Nile State were enrolled at delivery time in a cross-sectional study conducted between Jan 2012-Dec 2015. Malaria infection in the collected maternal peripheral, placental, umbilical cord samples was determined microscopically, and ELISA was used to measure the plasma levels IL-4, IL-6, IL-10, IL-17A, and INF γ in the collected positive and negative malaria samples.

**Results:**

Elevated levels of IL-4 and IL-10 and reduced levels of IL-6 were detected in the malaria positive samples in comparison to the negative ones in the three types of the samples investigated. Maternal, IL-4 and IL-10 were significantly higher in the samples collected from the PM infected group compared to the non-infected control (P < 0.001). While the absence of PM was significantly associated with the IL-6 and maternal IFN-γ levels, maternal IL-17A, placental and umbilical cord IFN-γ levels showed no significant difference (P = 0.214, P = 0.065, P = 0.536, respectively) due to infection. Haemoglobin level and birth weight were increased in the group with high levels of IL-6 and IL-17A, but not in the group with IL-4 and IL-10 levels. While significantly negative correlation was found between IFN-γ levels and birth weight for all three types of samples, only maternal peripheral IFN-γ level was significantly positively correlated with maternal haemoglobin (r = 0.171, P = 0.020).

**Conclusion:**

These results suggest that PM induces mother’s immune response and impairs her cytokine profile, which might alter maternal haemoglobin levels and the baby's birth weight.

## Background

*Plasmodium falciparum* infections during pregnancy result in pregnancy-associated malaria (PAM) and placental malaria (PM). In PM, the parasite ligand VAR2CSA mediates adhesion of infected erythrocytes (IEs) to chondroitin sulphate A CSA in the placental syncytiotrophoblast [1, 2]. Accumulation of IES in the placenta induces pathological changes that alter the materno-fetal exchange system and can lead to maternal morbidity and severe fetal and neonatal complications [3, 4]. While pregnancy is characterized by an induced immunosuppression [5], an activated immune system during pregnancy might have a role in protection against malaria and poor delivery outcomes [6, 7], and it might decrease the risk of both *P. falciparum* and *Plasmodium vivax* infections [8].

It is also known that *P. falciparum* IEs induce inflammation by monocytic infiltration of the malaria infected placenta, which is associated with maternal anaemia and low birth weight (LBW) [9, 10]. This inflammation may influence cellular functions by altering the balance of cytokines and chemokines in the peripheral and placental blood of the women [11, 12], and some cytokines can help resolve infections while others may also contribute to pathology [13–15]. Although the balance between pro- and anti-inflammatory cytokines is a key factor in the regulation of an effective immune response to PM [16], the roles of the produced cytokines are controversial. Among the cytokines that are produced, IL-27 and IL-6 can elicit both pro-inflammatory and anti-inflammatory effects, and high concentrations of IL-6 may protect against PM [17].

IFN-γ and IL-17A levels have been found to be negatively correlated with PM infection and maternal anaemia [15], and an association of IFN-γ with protection from malaria has been reported [18, 19]. Increased Th1 response due to infection was found to be incompatible with a successful pregnancy in mice [20]. An elevated level of IFN-γ may result in trophoblast damage, preterm delivery, and LBW associated with PM [21–23]. IL-17A may facilitate local inflammation by recruiting and activating immune cells, leading to the upregulation of inflammatory cytokine production [24], but little is known about its role in malaria infection during pregnancy and PM.

*Plasmodium falciparum* infection during pregnancy increases the placental levels of IL-10 and IL-4 [13, 25]. Elevated levels of the anti-inflammatory cytokine IL-10 in placental plasma were associated with PM, and were implicated in the pathogenesis of severe anaemia [9, 12, 13]. Moreover, IL-10 has been identified as an immunosuppressive cytokine associated with poor pregnancy outcome in a mouse model of PM [26]. Furthermore, placental infection influences fetal immune responses by affecting the Th1/Th2 balance in umbilical cord blood through IL-10 production by T regulatory cells as a result of infection [27].

An understanding of the inflammatory responses in the mother and foetus and the cause of LBW during PM could help optimize efforts to prevent the consequence of placental sequestration that results in poor birth outcomes.

This study aims to investigate the inflammatory environment in maternal peripheral, placental, and umbilical cord blood in response to PM and the extent to which this may influence pregnancy outcomes. The study was conducted with a cohort of Sudanese women from the Blue Nile State, which has a high rate of seasonal malaria transmission.

## Methods

### Study area and population

This cross-sectional study consisted of a cohort of pregnant women in Blue Nile State, Sudan at their time of delivery, who were recruited between January 2012 and December 2015. The study area, population, and the sample collection method have been described elsewhere [28]. Briefly, a total of 1149 consenting pregnant women were recruited for assessment of the prevalence and risk factors of PM in the study area. The study participants for the sub-study described in this paper included a group of 185 women for whom at least 2 ml of peripheral, placental and cord plasma were available and they signed additional consent for the immune response investigation. Information on the mother's socio-demographic characteristics, such as age, parity, education, use of bed nets and anti-malarial drugs, was obtained via a questionnaire. The haemoglobin level was assessed, and maternal anaemia at delivery was defined as a haemoglobin level < 11 g/d. The neonates were weighed immediately after birth.

### Sample collection

Maternal peripheral, placental and umbilical cord blood samples were collected immediately after delivery in heparinized vacutainer tubes. A portion of each sample was used to prepare smears for malaria microscopy and to determine the haemoglobin levels in maternal blood. The rest of the blood was centrifuged at 3000 g for 3 min, and the undiluted plasma was aliquoted into several tubes (to avoid freeze and thaw) and stored at −80 °C until thawed on the day that cytokine assays were performed. The blood smears were routinely stained with Giemsa and microscopically examined by two expert microscopists to determine the presence of malaria parasites.

### Measurement of the cytokines

The plasma of maternal peripheral, placental and neonate umbilical cord blood was simultaneously subjected to cytokine screening of IL-4, IL-6, IL-10, IL-17A and INF γ using Human ELISA MAX™ Deluxe commercial kits (BioLegend, USA) according to the manufacturer's instructions. The sensitivity of the assay for each cytokine was 2 pg/ml, which was the minimum detectable concentration of each cytokine.

### Statistical analysis

Data were analysed using SPSS software for Windows. Continuous data were checked for normality using the Shapiro–Wilk test; they were expressed as median (interquartile) if they were found to not be normally distributed. The Mann–Whitney U test and the Kruskal–Wallis H test (non-parametric) were used to compare the non-normally distributed continuous variables between two and three groups, respectively. Spearman's correlation test was used to assess the correlations between the non-normally distributed continuous variables.

## Results

### General characteristics

A total of 185 samples were collected at delivery time from maternal peripheral, placental, and umbilical cord blood. The median (interquartile) of the age was 20 (18−22 years). The median (interquartile) of the parity was 2 (1−3). The median (interquartile) of the haemoglobin level of the participants was 10.2 (9.3−11.3) g/dl. A total of 127 (68.6%) women had anaemia (haemoglobin < 11.0 g/dl). Ninety-two (49.7%) of the samples from the maternal peripheral blood, 97(52.4%) of the placental and 55 (29.7%), the umbilical cord was blood film positive for *P. falciparum* infection. Forty-three (23.2%) of the investigated blood films were positive in the three compartments of the same patient.

The median (interquartile) of the birth weight was 2.4 kg (2.3−2.7). Ninety-eight (53.0%) of the new-borns had LBW (birth weight < 2.5 kg).

### Cytokines levels in the maternal, placental and umbilical cord samples

With the exception of IL-17A levels, the cytokines levels were significantly lower in the umbilical cord samples than the maternal peripheral and placental samples. IL-17A level was similar in the maternal and umbilical cord, however the maternal and umbilical cord level was significantly lower compared with the placental level (Table 1).

**Table 1:** Comparisons of median cytokine levels (interquartile range) in the maternal, placental and cord blood compartments of women positive and negative for malaria infections

Variables		Maternal	Placental	Umbilical cord	P
Total number		185	185	185	
Number of malaria positive		92	97	55	
Number of malaria negative		93	88	130	
IL4	Total	4.2 (2.3−7.3)	6.7 (4.4−10.6)	1.9 (1.2−2.9)	< 0.001
	Malaria positive	6.5 (3.9−8.5)	10.3 (8.3−13.3)	2.5 (1.8−3.6)	< 0.001
	Malaria negative	3.1 (1.7−4.3)	4.7 (3.4−6.0)	1.6 (1.0−2.5)	< 0.001
	P value	< 0.001	< 0.001	< 0.001	
IL 6	Total	39.2 (23.7−63.4)	19.8 (12.3−30.6)	8.3 (4.1−14.2)	< 0.001
	Malaria positive	27.6 (20.8−40.9)	16.1 (9.4−20.1)	5.6 (2.8−11.6)	< 0.001
	Malaria negative	58.3 (38.1−73.2)	29.4 (19.4−41.1)	9.5 (5.5−14.5)	< 0.001
	P value	< 0.001	< 0.001	0.002	
IL10	Total	63.5 (31.5−102.0)	37.3 (17.9−71.5)	12.6 (7.5−25.3)	< 0.001
	Malaria positive	89.4 (49.5−175.5)	65.4 (36.2−102.5)	15.6 (9.4−37.8)	< 0.001
	Malaria negative	46.5 (24.4−72.0)	19.4 (12.7−37.0)	13.5 (6.8−20.9)	< 0.001
	P value	< 0.001	< 0.001	0.032	
IL17A	Total	3.2 (1.4−5.4)	8.6 (6.4−14.2)	3.3 (2.1−7.0)	< 0.001
	Malaria positive	2.5 (1.4−5.2)	7.5 (5.6−8.6)	2.8 (2.0−3.5)	< 0.001
	Malaria negative	3.6 (1.4−5.6)	14.3 (10.0−16.3)	3.6 (2.3−8.0)	< 0.001
	P value	0.214	< 0.001	0.002	
IFN-γ	Total	68.1 (39.1−107.0)	86.7 (45.9−192.7)	27.6 (18.4−67.8)	< 0.001
	Malaria positive	48.8 (32.9−78.0)	129.9 (36.8−281.9)	32.1 (17.2−96.3)	< 0.001
	Malaria negative	97.2 (58.3 − 121.9)	76.1 (49.6 − 112.1)	27.5 (18.6−57.5)	< 0.001
	P value	< 0.001	0.065	0.536	

### Cytokines levels and malaria infections

While the levels (in three compartments) of IL- 4 and IL-10 were significantly higher, the levels of IL-6 in all the compartments and maternal IFN-γ were significantly lower in the PM positive samples in comparison to the PM negative samples. Maternal peripheral IL-17A levels and the placental and umbilical IFN-γ levels showed no significant difference between the PM infected group and the non-infected group (Table 1).

### Cytokines levels and parity

There was no significant difference in the maternal peripheral and placental IL-17A levels between the primiparous women and the parous women. The level of IL-4 was significantly higher in the primiparous women in all three types of samples (maternal peripheral, placental, and umbilical cord blood). The IL-6 levels in the maternal peripheral, placental and umbilical cord blood, and the umbilical cord levels of IL-17A were significantly lower in the primiparous women. The maternal peripheral and placental IL-10 levels and the placental and umbilical cord IFN-γ levels (no significant difference in the maternal peripheral IFN-γ levels) were significantly higher in the primiparous women in comparison to the parous women (Table 2).

**Table 2:** Comparison of cytokines level between primiparous and parous women

Variables		Primiparaous (n = 92)	Parous (93)	P
IL4	Maternal	4.6 (3.2−8.1)	3.7 (1.8−5.7)	0.001
	Placental	7.5 (5.2−12.5)	6.2 (4.2−9.7)	0.014
	Umbilical cord	2.0 (1.2−3.0)	1.6 (1.0−2.6)	0.03
IL 6	Maternal	33.2 (31.6−57.5)	43.9 (29.7−68.8)	0.007
	Placental	16.5 (10.2−27.7)	22.3 (16.9−34.9)	0.005
	Umbilical cord	7.0 (3.7−12.3)	9.6 (4.6−15.6)	0.050
IL10	Maternal	70.2 (40.5−129.5)	51.3 (27.6−89.7)	0.005
	Placental	45.2 (19.7−92.0)	32.2 (16.4−58.1)	0.022
	Umbilical cord	14.3 (8.4−28.7)	11.0 (7.3−18.9)	0.077
IL17A	Maternal	3.3 (1.3−5.9)	2.8 (1.4−5.1)	0.724
	Placental	8.7 (5.9−14.3)	8.6 (6.4−14.0)	0.743
	Umbilical cord	3.1 (1.5−6.9)	3.6 (2.5−7.1)	0.027
IFN-γ	Maternal	63.7 (34.3−98.3)	73.3 (44.5−111.5)	0.231
	Placental	180.9 (58.4−292.7)	59.3 (37.7−87.8)	< 0.001
	Umbilical cord	37.3 (19.7−96.3)	23.7 (19.5−48.7)	0.005

### Correlations between cytokines

The IL-4 levels in the plasma of the three types of blood samples were significantly negatively correlated with the IL-6 levels, and they were positively correlated with the IL-10 levels. There was a borderline negative correlation between the maternal peripheral IL-4 levels and the maternal peripheral IFN-γ levels. A negative correlation and a positive correlation were found between the placental IL-4 levels and the placental IL-17A levels and the IFN-γ level, respectively. In the umbilical cord samples, the level of IL-4 was negatively correlated with the level of IL-17A.

The IL-6 levels in all three types of the blood samples were significantly negatively correlated with the levels of IL-10 and positively correlated with the levels of IL-17A. The maternal and placenta IL-6 levels were positively and negatively, respectively correlated with maternal and placenta IFN-γ.

While no correlation was found between the maternal peripheral level of IL-10 and the maternal peripheral levels of IL-17A and IFN-γ, negative correlations were found between the placental and umbilical cord levels of IL-10 and IL-17A, and a positive correlation was found between IL-10 levels and the level of placental and umbilical cord of IFN-γ. No correlation was found between the level of IL-17A for the maternal peripheral, placental and umbilical cord blood samples and the IFN-γ levels of the plasma in the three types of samples.

### Correlations between cytokines, maternal haemoglobin and birthweight

Maternal haemoglobin was significantly negatively correlated with the levels of IL-4 and IL-10 in the maternal peripheral, placental and umbilical cord blood samples; however, no significant correlation was found between maternal haemoglobin and the level of the peripheral IL-17A. Significantly positive correlation and negative correlations were found between the levels of IL-6 and IFN-γ, respectively, and birth weight for all three types of samples, which was significantly negatively correlated with the levels of, IL-4 and IL-10. While no significant correlation was found between birth weight and maternal levels of IL-17A, it was significantly positively correlated with the levels of IL-17A in the placental and umbilical cord samples (Tables 3, 4, 5).

**Table 3:** Correlations between maternal plasma levels of cytokines, maternal hemoglobin and birth weight

Variables	Hemoglobin	Birth weight	IL4	IL 6	IL10	IL17A	IFN-γ
	*r*	*r*	*r*	*r*	*r*	*r*	*r*
	*P*	*P*	*P*	*P*	*P*	*P*	*P*
Hemoglobin	*1.000*	0.749	−0.544	0.430	− 0.478	0.130	0.171
		< 0.001	< 0.001	< 0.001	< 0.001	0.078	0.020
Birth weight	0.749	*1.000*	− 0.525	0.455	− 0.484	0.129	− 0.233
	< 0.001		< 0.001	< 0.001	< 0.001	0.080	0.001
IL4	− 0.544	− 0.525	*1.000*	− 0.476	0.438	0.034	− 0.142
	< 0.001	< 0.001		< 0.001	< 0.001	0.647	0.054
IL 6	0.430	0.455	− 0.476	*1.000*	− 0.449	0.151	0.204
	< 0.001	< 0.001	< 0.001		< 0.001	0.040	0.005
IL10	− 0.478	− 0.484	0.438	− 0.449	*1.000*	–0.110	− 0.056
	< 0.001	< 0.001	< 0.001	< 0.001		0.135	0.448
IL17A	0.130	0.129	0.034	0.151	− 0.110	*1.000*	0.063
	0.078	0.080	0.647	0.040	0.135		0.395
IFN-γ	0.171	− 0.233	− 0.142	0.204	− 0.056	0.063	*1.000*
	0.020	0.001	0.054	0.005	0.448	0.395

**Table 4:** Correlations between placental plasma levels of cytokines, maternal hemoglobin and birth weight

Variables	Hemoglobin	Birth weight	IL4	IL 6	IL10	IL17A	IFN-γ
	*r*	*r*	*r*	*r*	*r*	*r*	*r*
	*P*	*P*	*P*	*P*	*P*	*P*	*P*
Hemoglobin	*1.000*	0.749	− 0.586	0.403	− 0.436	0.377	− 0.146
		< 0.001	< 0.001	< 0.001	< 0.001	< 0.001	0.047
Birth weight	0.749	*1.000*	− 0.574	0.402	− 0.445	0.391	− 0.146
	< 0.001		< 0.001	< 0.001	< 0.001	< 0.001	0.038
IL4	− 0.586	− 0.574	*1.000*	− 0.406	0.462	− 0.357	0.185
	< 0.001	< 0.001		< 0.001	< 0.001	< 0.001	0.012
IL 6	0.403	0.402	− 0.406	*1.000*	− 0.393	0.335	− 0.199
	< 0.001	< 0.001	< 0.001		< 0.001	< 0.001	0.007
IL10	− 0.436	− 0.445	0.462	− 0.393	*1.000*	− 0.446	0.299
	< 0.001	< 0.001	< 0.001	< 0.001		< 0.001	< 0.001
IL17A	0.377	0.391	− 0.357	0.335	− 0.446	*1.000*	0.117
	< 0.001	< 0.001	< 0.001	< 0.001	< 0.001		0.114
IFN-γ	− 0.146	–0.146	0.185	− 0.199	0.299	− 0.117	*1.000*
	0.047	0.048	0.012	0.007	< 0.001	0.114

**Table 5:** Correlations between umbilical cord plasma levels of cytokines, maternal hemoglobin and birth weight

Variables	Hemoglobin	Birth weight	IL4	IL 6	IL10	IL17A	IFN-γ
	*r*	*r*	*r*	*r*	*r*	*r*	*r*
	*P*	*P*	*P*	*P*	*P*	*P*	*P*
Hemoglobin	*1.000*	0.749	− 0.512	0.334	− 0.030	0.460	− 0.073
		< 0.001	< 0.001	< 0.001	< 0.001	< 0.001	0.322
Birth weight	0.749	*1.000*	− 0.493	0.370	− 0.329	0.500	− 0.069
	< 0.001		< 0.001	< 0.001	< 0.001	< 0.001	0.035
IL4	− 0.512	− 0.493	*1.000*	− 0.316	0.294	− 0.304	0.140
	< 0.001	< 0.001		< 0.001	< 0.001	< 0.001	0.058
IL 6	0.334	0.370	− 0.316	*1.000*	− 0.248	0.333	− 0.024
	< 0.001	< 0.001	< 0.001		0.001	< 0.001	0.741
IL10	− 0.300	− 0.329	0.294	− 0.248	*1.000*	− 0.324	0.215
	< 0.001	< 0.001	< 0.001	0.001		< 0.001	0.003
IL17A	0.460	0.500	− 0.304	0.333	− 0.324	*1.000*	− 0.136
	< 0.001	< 0.001	< 0.001	< 0.001	< 0.001		0.065
IFN-γ	− 0.073	− 0.069	0.140	− 0.024	0.215	− 0.136	*1.000*
	0.322	0.035	0.058	0.741	0.003	0.065	

## Discussion

The present study found that malaria infection altered the investigated cytokines (IL-4, IL-6, IL-10, IL-17A and INF-γ) in Sudanese maternal, placental and neonatal plasma collected from Blue Nile State. Disturbance of cytokines secretions has been linked to many pathological disorders including poor pregnancy outcomes [15].

In the current study, the levels of IL-4 and IL-10 were elevated but the levels of IL-6 and IL-17 were reduced in the plasma of the maternal peripheral, placental and umbilical cord blood samples of the PM infected mothers in comparison to the non-infected group. Moreover, the maternal peripheral level of IL-17A and the placental and neonate IFN-γ levels were not significantly different due to the infection.

Several previous studies have shown elevated levels of type 2 anti-inflammatory cytokines IL-10 and IL-4 in the malaria-infected group [12, 13, 15, 23, 25, 29, 30]. Conversely, the present results were different from the previous findings in an area with unusable malaria transmission in Sudan, where higher levels of IL-4 and IL-10 in the non-infected group was reported [11]. The findings of the increased IL-6 levels in non-infected women is in agreement with the results of other studies [11, 16, 31, 32].

Interestingly, the level of cytokines in the maternal peripheral samples among the investigated groups was significantly correlated with the levels in the placental and umbilical cord samples. Nevertheless, plasma from the umbilical cord (except for the maternal peripheral IL-17A levels) contained significantly lower concentrations of the investigated cytokines in comparison to the peripheral and placental plasma in both study groups (PM infected and non-infected groups). Kabyemela et al. [25], in Tanzania, and Ibitokou [31], in Benin, reported similar levels of IFN-γ between the investigated groups. The results of the present study corroborate the findings of a previous study that reported higher levels of IFN-γ in infected placentas [9], and a study from Cameron that found an increase in maternal IFN-γ and IL-17-A levels in non-infected controls [15]. The elevated detection levels of IFN-γ in the investigated samples may be due to the ability of the innate immune response to produce IFN-γ to clear the parasite, probably as the first line of defense against both peripheral and placental infection [18]. It has been suggested that the differences in cytokine responses associated with malaria infection have a gravidity-based pattern [22].

Infiltration of immune cells in the placental intervillous spaces, due to the sequestration of *P falciparum* IEs, disrupting Th1 and Th2 cytokines balance in both placental and peripheral blood [22, 33]. Contradictory findings about the role of impaired cellular immune responses directed against malaria parasites in pregnancy and pathogenesis of PM have been described [12, 13, 15, 17, 21, 25, 34, 35]. However, the differences in the cytokine profiles between these studies may be attributed to variations in the cytokine measurement methods used and the malaria endemicity in different study areas, as well as differences in the diagnostic techniques and the study population.

In the present study, among the cytokines assessed, IL-4 and IL-10 were elevated and significantly correlated (P < 0.001) in the plasma of the three types of blood samples that were investigated; no correlation was found between the maternal peripheral levels of IL-10 and IFN-γ (P = 0.448). IL-17A was positively correlated with IL-6, but no correlation was found between the IFN-γ in the plasma of the three types of samples. IFN-γ and IL-17-A are both produced by effector helper T cells, and IL-17A can facilitate regulation of inflammatory cytokine production by accelerating specific inflammation via the recruitment and activation of immune cells. IL-6 is both a target of IL-17 and a differentiation factor for Th17 cells that can led to an increase in IL-17 [36]. Agudelo et al. [23] observed a significant correlation between IL-4 and IL-10 in the placental samples, but not in the peripheral blood, and levels of maternal IL-10 and IFN-γ were positively correlated. Moreover, Agudelo et al. [23] reported high expression of IFN-γ, TNF and IL-10 in the placental tissues and peripheral blood samples, and placental IL-4 in the infected women in comparison to the non-infected group. Due to the high expression associated with PM infection, it has been suggested that the IL-10 level in peripheral blood to be used as biomarker of placental inflammation related to PM [25] or as an immunosuppressive factor by decreasing anti-parasitic cellular immune responses [26].

The present study found that IL-6 and IFN-γ were associated birth weight and the maternal haemoglobin level, accompanied by the negative correlation of IL-4 and IL-10 in the plasma of the three types of samples investigated. It has been documented that there is a disturbance of cytokine equilibrium in malaria during pregnancy, and PM may be involved in many pathological disorders; it may also have negative consequences, such as LBW and reduced maternal haemoglobin level [37–39].

While the levels of IL-17A in the placental and umbilical cord samples were significantly positively correlated with birth weight and maternal haemoglobin, no significant correlation was found with the IL-17A levels in the maternal peripheral samples. The roles of IFN-γ and IL-10 in the malaria-infected women with maternal anaemia and baby birth weight was controversially documented in a reviewed by Seitz et al*.* [39]. Although Djontu et al*.* [16] reported no significant association between IL-6 level, maternal haemoglobin and baby birth weight, an elevated level of IL-6 was associated with anaemia in another study [29].

Similar to the current study’s findings, an association was reported between maternal haemoglobin and IL-17A levels and the peripheral plasma level of IFN-γ. It has been suggested that both cytokines provide protection against infection [15]. Furthermore, elevated levels of IL-17 with high levels of IL-4, IL-12 and IFN-γ were associated with haemoglobin loss in malaria recovered semi-immune mice [40]. Fitri et al. [41] reported that an imbalance between IL-17 and IL- 10 caused low fetal weight in *Plasmodium berghei* infection in mice.

Disturbance of pro-inflammatory cytokines and the inflammatory disorder of iron haemostasis led to the development of malarial anaemia [42]. Elevated levels of circulating IL-6, which play a vital role in T cells differentiation and immune response polarization, have been strongly related to reduced haemoglobin concentration in reticulocytes.

A limitation of the current study is that it did not check for the presence of other infections which could influence variations in cytokine levels as the study was conducted in a setting of availability of other common infections during pregnancy.

## Conclusion

In the present study, maternal peripheral infection and PM induces mother’s immune response accompanied by secretion of various cytokines in the maternal peripheral, placental and umbilical cord blood in Sudanese women. The present findings support the evidence reported in previous studies, which found that PM affects cytokines levels in infected women. However, longitudinal studies are needed to understand the maternal immune response throughout the entire course of pregnancy.

## Data Availability

The datasets used and/or analysed during the current study are available from the corresponding author on reasonable request.
